# High Figure‐of‐Merit Telluride‐Based Flexible Thermoelectric Films through Interfacial Modification via Millisecond Photonic‐Curing for Fully Printed Thermoelectric Generators

**DOI:** 10.1002/advs.202202411

**Published:** 2022-09-14

**Authors:** Md Mofasser Mallick, Leonard Franke, Andres Georg Rösch, Holger Geßwein, Zhongmin Long, Yolita M. Eggeler, Uli Lemmer

**Affiliations:** ^1^ Light Technology Institute Karlsruhe Institute of Technology 76131 Karlsruhe Germany; ^2^ Institute for Applied Materials Karlsruhe Institute of Technology 76344 Eggenstein‐Leopoldshafen Germany; ^3^ Laboratory for Electron Microscopy Karlsruhe Institute of Technology 76131 Karlsruhe Germany; ^4^ Institute of Microstructure Technology Karlsruhe Institute of Technology 76344 Eggenstein‐Leopoldshafen Germany

**Keywords:** flexible films, high figure‐of‐merit, photonic curing, printed thermoelectrics

## Abstract

The thermoelectric generator (TEG) shows great promise for energy harvesting and waste heat recovery applications. Cost barriers for this technology could be overcome by using printing technologies. However, the development of thermoelectric (TE) materials that combine printability, high‐efficiency, and mechanical flexibility is a serious challenge. Here, flexible (SbBi)_2_(TeSe)_3_‐based screen‐printed TE films exhibiting record‐high figure of merits (ZT) and power factors are reported. A high power factor of 24 µW cm^−1^ K^−2^ (ZT_max_ ≈ 1.45) for a p‐type film and a power factor of 10.5 µW cm^−1^ K^−2^ (ZT_max_ ≈ 0.75) for an n‐type film are achieved. The TE inks, comprised of p‐Bi_0.5_Sb_1.5_Te_3_ (BST)/n‐Bi_2_Te_2.7_Se_0.3_ (BT) and a Cu‐Se‐based inorganic binder (IB), are prepared by a one‐pot synthesis process. The TE inks are printed on different substrates and sintered using photonic‐curing leading to the formation of a highly conducting *β*‐Cu_2−_ *_*δ*_* Se phase that connects “microsolders,” the grains resulting in high‐performance. Folded TEGs (f‐TEGs) are fabricated using the materials. A half‐millimeter thick f‐TEG exhibits an open‐circuit voltage (*V*_OC_) of 203 mV with a maximum power density (*p*_max_) of 5.1 W m^−2^ at ∆*T* = 68 K. This result signifies that a few millimeters thick f‐TEG could power Internet‐of‐Things (IoTs) devices converting low‐grade heat to electricity.

## Graphical abstract

A “microsoldering” technique is reported through millisecond photonic curing to prepare a pair of flexible p‐type Bi_0.5_Sb_1.5_Te_3_ (p‐BST) and n‐type Bi_2_Te_2.7_Se_0.3_ (n‐BT) thermoelectric films with bulk‐like performance for fully printed thermoelectric generators. A Cu‐Se‐based inorganic binder is used for soldering p‐BST/n‐BT grains through millisecond‐photonic‐curing. Two major challenges in printed thermoelectrics are overcome: a) high interfacial resistance and b) nonflexibility.

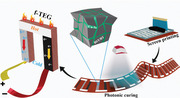

## 1 Introduction

Energy in different forms is fated mostly to end as waste heat. Approximately 65% of primary energy is released as waste heat after utilization in modern societies.^[^ ^1^ ^]^ The conversion of this enormous amount of leftover waste heat into useful electricity could contribute to the renewable energy sector and fight climate change. The thermoelectric generator (TEG) is one of the straightforward technologies which can convert waste heat into useful electricity.^[^ ^2^ ^]^ Apart from waste heat recovery, the TEG can also harvest low‐grade heat such as body heat for different internet‐of‐things (IoTs) applications.^[^ ^3^ ^]^ The goodness of thermoelectric (TE) materials used in a TEG is defined by the figure‐of‐merit, *ZT* = *S* ^2^ *σT*/*κ*, where *σ, S*, and *κ* are electrical conductivity, the Seebeck coefficient, and thermal conductivity of the materials.^[^ ^4^ ^]^ Despite remarkable progress in high‐performance materials development, several challenges have made TE technology so far less successful than other energy conversion technologies like photovoltaics.^[^ ^5^ ^]^ The availability of enormous waste heat and the progress in materials science have been motivating the research community for decades; unfortunately, they are yet to make a significant contribution in the renewable sector.^[^ ^6^ ^]^ It is difficult to fabricate a TEG that yields a power output according to the ZT values of the device materials. The high electrical and thermal contact resistance are two of the major problems along with the complex manufacturing process, which considerably lowers the performance of TEGs.^[^ ^7^ ^]^ Therefore, the high manufacturing cost per output power still hinders the application of state‐of‐the‐art bulk thermoelectrics. In addition, many of the potential TE applications areas deal with nonflat surfaces such as exhaust systems of vehicles, heat exchangers, cylindrical pipes with hot liquids, and human skin.^[^ ^8^ ^]^ The conventional bulk thermoelectrics does not offer shape‐conformity; hence, the bulk TEGs do not function effectively for these applications due to the poor thermal coupling. The synergy between thermoelectrics and printing technology could effectively overcome the difficulties associated with bulk TEGs.^[^ ^9^ ^]^ Consequently, research on printed thermoelectrics is gaining significant momentum.^[^ ^10^, ^11^ ^]^ However, it remains just a scientific ambition, as it is not easy to overcome the entanglement of printability, high performance, and flexibility. The well‐known conducting polymers‐based printed organic materials possess good printability and flexibility but low TE performance.^[^ ^12^ ^]^ In inorganic‐based printed TE materials, the organic binders, solvents, and additives increase the interfacial resistance at the grain boundaries affecting the electrical conductivity *σ*, which leads to low performance.^[^ ^13^ ^]^ Furthermore, inorganic‐based printed TE materials are generally coarse and do not show good flexibility. Apart from high performance, printability and good flexibility are essential to achieve low‐cost manufacturing and application of printed TEGs. However, developing a high‐performance printed TE material has been challenging, making it flexible even a bigger challenge. The (Sb/Bi)_2_(TeSe)_3_ based p‐ or n‐type alloys are notable TE materials for bulk device applications for their high room temperature (RT) performance.^[^ ^14^, ^15^ ^]^ Hence, they have been targeted for printed thermoelectrics widely.^[^ ^16^, ^17^, ^18^, ^19^, ^20^, ^21^ ^]^ The (SbBi)_2_(TeSe)_3_ based printed TE materials have been developed using different printing techniques such as screen printing, inkjet printing, and dispenser printing.^[^ ^22^, ^23^, ^24^, ^25^, ^26^, ^27^, ^28^ ^]^ Unfortunately, the organic ingredients interrupt the charge transport across the grain boundaries in printed films lowering the ZT. In addition, the material processing involves postprinting pressure‐treatment or high‐temperature annealing to achieve high‐performance, which requires expensive high‐temperature stable substrates and is not feasible for large‐scale manufacturing such as Roll‐to‐Roll printing. Apart from the (SbBi)_2_(TeSe)_3_ based printed TE materials, other high‐performance chalcogenides printed films are reported with or without a postprinting pressure treatment.^[^ ^29^, ^30^, ^31^ ^]^ However, the flexibility and robustness required for large‐scale manufacturing of printed TEGs have not been achieved. Furthermore, the development of a pair of p‐ and n‐type high‐performance TE films with a similar synthesis routine is also essential for the large‐scale manufacturing of efficient printed TEGs.

**Figure 1:**
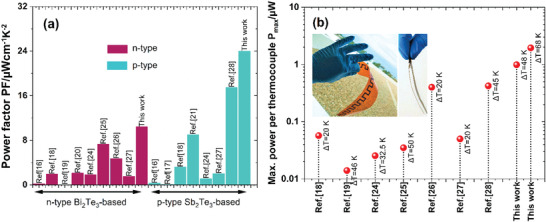
a) Maximum power factor of TE legs and b) the device power‐output per thermocouple of recently reported pressure treatment‐free printed (SbBi)_2_(TeSe)_3_ based TEGs.

This work reports a grain “microsoldering” technique through millisecond photonic‐curing, replacing the traditional sintering process to manufacture a pair of flexible p‐type Bi_0.5_Sb_1.5_Te_3_ (p‐BST)‐ and n‐type Bi_2_Te_2.7_Se_0.3_ (n‐BT)‐based TE films with bulk‐like high performance for printed device applications. We have used a Cu‐Se‐based inorganic binder (IB) as soldering material to connect p‐BST/n‐BT grains through millisecond‐photonic‐curing. The photonic sintering process protects the low‐temperature flexible substrates like polyethylene naphthalate (PEN), polyethylene terephthalate (PET) from damage. Consequently, two major challenges in printed thermoelectrics are overcome: a) high grain interfacial resistance and b) nonflexibility in a printed TE film. The Cu‐Se‐based IB binder reduces the grain interfacial resistance between the microparticles, enhancing the TE performance. The ball‐milling combined with photonic curing improves printability and film flexibility. In addition, the photonic‐curing process has reduced the sintering time from several hours to a few milliseconds. The mechanism of the photonic‐curing technology is discussed in the next section in detail. Using the developed p‐ and n‐type TE films, printed folded TEGs (f‐TEGs) have been fabricated on flexible substrates for the demonstration. The comparison studies of the TE films and f‐TEGs performances are shown in **Figure** **1**.

## 2 Photonic‐Curing Process and Parameters for p‐BST/n‐BT Films on Different Substrates

The photonic curing technology is employed to sinter a thin film using a xenon flash lamp, thus avoiding the significant rise of substrate temperature.^[^ ^32^, ^33^ ^]^ The two main factors that control the film's sintering temperature are the widths and amplitudes of the pulses in the pulse train emitted by the high‐power flash lamp.^[^ ^34^, ^35^ ^]^ A current passes through the flash lamp due to a voltage (*V*_P_) to gain high intensity and a broad radiation spectrum. The temperature of the film increases through the absorption of the photons to fuse the particles. The film temperature rapidly decreases without a substantial change in substrate temperature after turning off the flash lamp. Hence, this technology allows different functional films to be sintered on low‐temperature substrates such as PET, PEN, and paper. Furthermore, as the photosintering process is very fast, it minimizes the oxidization of the films even when sintered in the air. Therefore, due to the low‐cost substrates and the quick sintering process, photonic curing technology could significantly reduce the production cost of printed functional devices, including printed TE devices. In the following, we elaborate on the underlying principles of photonic‐curing of TE films on a substrate.

The photonic curing process involves three main steps: a) absorption of light (photon), b) sintering of the film, and c) rapid cooling of the sintered film. The photonic curing process is modeled using a computational fluid dynamics program considering; i) radiative heating of the film, ii) convective cooling from the top surface, and iii) the conductive cooling from the bottom surface of the film. The results help to estimate the temperature of the sintered films and the substrates during the photonic sintering process.

Optimum parameters are chosen for the curing process, depending on the type and dimension of the materials and substrates. Considering most of the flash radiation is absorbed by the film, the maximum temperature of the surface can be estimated using Stefan's law, $T\;=\sqrt[{4}]{R_{T}/\sigma_{T}}\;$, where *T* is the temperature of the film, *R*_T_ is the energy flux on the surface, and *σ*_T_ is the Stefan‐Boltzmann constant.^[^ ^36^ ^]^ The air temperature far from the film surface and the heat transfer coefficient between the film and air are required to model convective cooling. The air temperature is considered to be at room temperature, and the heat transfer coefficient *(h)* is calculated using the Nusselt number *(Nu_l__l_*), thermal conductivity *(κ)*, and characteristic length of the film *(l)*. The *h* is defined as^[^ ^37^ ^]^

(1) $$h=\frac{Nu_{l}\kappa }{l}$$

The *Nu_l_* is determined using the Rayleigh number *(Ra_l_)* and the Prandtl number *(Pr_l_)* via the following equation

(2) $$Nu_{l}=0.68+\frac{0.67Ra_{l}^{1/4}}{{\left[1+{\left(0.492/Pr_{l}\right)}^{9/16}\right]}^{4/9}}$$

The *Pr_l_* is calculated using the specific heat (*C*_p_), the dynamic viscosity *(µ)* and *κ* ^37^ via

(3) $$Pr_{l}=\frac{C_{p}\mu }{\kappa }$$

The *Ra_l_* is the product of the Pr_*l*_ and the Grashof number (*Gr_l_*), *Ra_l_* = *Pr_l_* × *Gr_l_*. The *Gr_l_* is defined as

(4) $$Gr_{l}=\frac{g\beta \left(T_{s}-T_{\propto }\right)l^{3}}{v^{2}}$$

where *g* is the gravitational constant*, β* is the volumetric expansion coefficient, and *v* is the kinematic viscosity.^[^ ^37^ ^]^ The conductive cooling is modeled considering heat transfer through the solid slabs (film and substrate) using the thermal conductivity, specific heat, and substrate density. Hence, the temperature dynamics at different locations can be determined by the physical properties of the film and the substrate.

**Figure 2:**
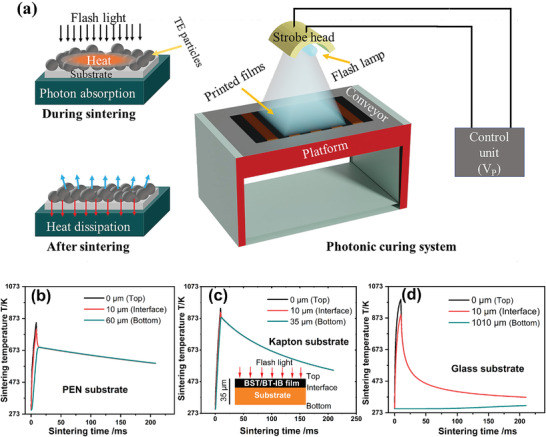
a) Schematic diagram of the photonic curing system and sintering process. Simulated temperature transients at different positions for p‐BST/n‐BT film on b) PEN, c) Kapton, and d) glass. The exposure of the respective sample to the flash lamp is less than 10 ms. The underlying model is described in the text. For the Kapton substrate, the temperature at the top of the film, at the interface, and the bottom of the substrate are similar due to its lower thickness (25 µm).

We have employed this model using the SimPulse numerical software to simulate the transient temperature of the (p‐BST/n‐BT)‐IB film on different substrates such as PEN, Glass, and Kapton. We have used the Novacentrix PulseForge 1200 photonic curing tool with a maximum radiant exposure of 22 J cm^2^ and a peak instantaneous power output of 4.3 kW cm^−2^. The maximum voltage of the flash lamp (*V*_P_) was 500 V. We have used a Cu‐Se‐based (IB) binder material to connect the p‐BST/n‐BT grains. A highly conducting *β*‐Cu_2_Se phase forms in the Cu‐Se‐based IB through photonic curing, which solders the p‐BST/n‐BT grains. As the *β*‐Cu_2_Se phase forms at *T* > 623 K.^[^ ^38^ ^]^ our target temperature on the film surface was above this. The upper‐temperature limit is determined by the film and substrate's thermodynamical and mechanical stability. Accordingly, curing parameters for different substrates were adjusted to reach the target temperature on the film surfaces. Simulated temperature profiles with the time for the (p‐BST/n‐BT)‐IB films on the PEN substrate (50 µm), Kapton (25 µm), and glass (1000 µm) are shown in **Figure** **2**b–d. The *V*_P_ = 330–350 V for the flexible substrate (PEN and Kapton) and 420 V for glass substrate with a sintering time of 8–10 ms were required to raise the sintering temperature to *T* > 623 K on the top and in the interface of the film. The film temperature reaches a higher value than the temperature at the bottom of the substrate, and it decreases rapidly. Hence, the substrate temperature rapidly drops below the softening temperature of the flexible substrates within a fraction of a second and damage is prevented. It can be observed that a higher *V*_P_ is required to reach 800 K for glass substrate as its thickness is more than 20 times higher than the flexible substrates. High substrate volume with higher thermal conductivity leads to fast dissipation of absorbed heat from the film. As a result, the temperature at the bottom of the glass substrate remains near RT.

## 3 Results and Discussion

### 3.1 Crystallographic, Mechanical, and Microstructural Properties of the Printed Films

**Figure 3:**
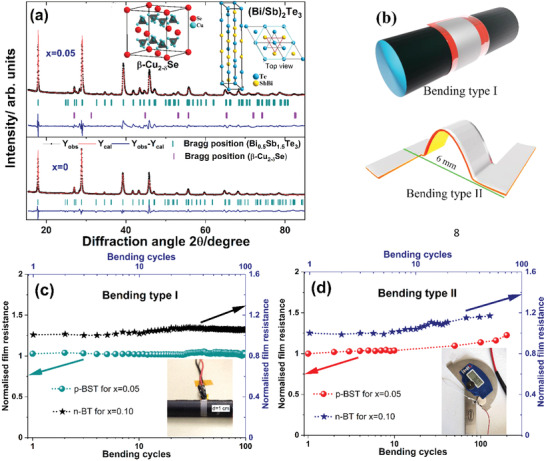
a) Room temperature XRD patterns with Rietveld refinement results of the films (1 − *x*)BST‐(*x*)IB for *x* = 0 and *x* = 0.05. The cubic *β*‐Cu_2−_ *_*δ*_* Se phase and trigonal p‐BST phase unit cells (inset). b) The printed films are bent during the bending test in two different ways. The change in normalized film resistance with bending cycles of the printed films for c) the bending type I and d) the bending type II.

The identification of crystallographic phases in the sintered films (1 − *x*)BST/BT‐(*x*)IB and their lattice structures were analyzed by Rietveld refinement using the FullProf program (see **Figure** **3**a and Figure S1, Supporting Information). The refinement result of the pristine films for *x* = 0 indicates a trigonal crystal structure belonging to the space group $R\bar{3}m$(. The X‐ray diffraction (XRD) patterns of the printed films for *x* > 0 correspond to a main p‐Bi_0.5_Sb_1.5_Te_3_/n‐Bi_2_Te_3_ phase with *β*‐Cu_2−_ *_*δ*_* Se as a secondary phase. The highly conducting cubic *β*‐Cu_2−_ *_*δ*_* Se phase belongs to the space group $Fm\bar{3}m$ is formed through the photonic curing via dissociative adsorption of elemental Se by Cu in the p‐ and n‐type printed films for *x* > 0. The *β*‐Cu_2−_ *_*δ*_* Se phase is found to be developed with a small fraction of excess Cu in a printed film containing elemental Se and Cu in a 2:1 molar ratio within 8–10 ms (see Figure S1c, Supporting Information). The detailed studies on the growth and transport properties of the *β*‐Cu_2−_ *_*δ*_* Se phase through milliseconds photonic curing and vacuum sintering have been reported in our previous report.^[^ ^38^, ^39^ ^]^ The lattice parameters of the p‐BST unit cell are estimated to be *a* = *b* = 0.418 (1) nm and *c* = 2.969 (4) where Bi/Sb atoms occupy Wyckoff position 6c (0, 0, 0.40) and the Te atoms occupies the Wyckoff positions 3a (0, 0, 0) and 6c (0, 0, 0.21). The Cu atoms exhibit disorder and are located in two distinct Wyckoff positions 8c (¼, ¼, ¼) and 32f (*x*, *x*, *x*), where the Se atoms are situated at the Wyckoff position 4a (0, 0, 0) in the cubic *β*‐Cu_2−_ *_*δ*_* Se lattice with unit cell parameter of 0.572 (9) nm. The calculated lattice parameters of p‐BST are slightly smaller than the reference values [Inorganic crystal structure database (ICSD) 184246; *a* = *b* = 0.43 nm, *c* = 3.06 nm]. It is found that the XRD peak of the TE phase shifts to a higher 2*θ* with increasing *x*. The shift was found to be ≈0.25° and ≈0.20° for p‐BST and n‐BT films respectively for *x* = 0.10 (see Figure S1, Supporting Information). Hence, the lattice parameters seem to be contracted slightly by the addition of IB while the crystal structures remain unchanged. The surfaces of the TE grains probably slightly modified due to the diffusion of excess Cu and Se atom. However, it was very difficult to locate the site of the diffused elements in the unit cell of the TE phase. Elemental line scan results also indicate that the elemental Cu and Se diffusion occurs during sintering.

The mechanical robustness of the printed TE elements is essential for large‐scale printed device production. They should withstand different mechanical forces during the manufacturing process and beyond to maintain their TE performance. Hence, the mechanical flexibility of the sintered films with *x* = 0.05 for p‐BST and with *x* = 0.10 for n‐BST used for device fabrication has been studied by bending it in two different ways several times (see Figure 3b–d). For bending type I, the bending test was performed by bending the films around a roller with a 10 mm diameter for 100 cycles (see Figure 3b,c). For bending type II, two sides of the sintered films with 12 mm length were attached to two arms of a digital protractor, as shown in Figure 3b,d. Before recording the data, the resistance of the films versus bending cycle, we calibrated the distance between two ends of the films with the angle between two legs of the protractor. There was no strain on the films when the angle was 49°. The angle was reduced to 30° to bend the films where the distance between the two ends of the films decreased to 6 from 12 mm. The changes in the film resistance (*R*_o_) are recorded with bending up to 200 cycles. No significant change in the film resistance is observed (see Figure 3c,d) for both the bending types. The normalized film resistance (*R*/*R*_o_) of the p‐BST and n‐BT films remains <1.08 for bending type I, and the same remains <1.16 for bending type II. The results signify the robustness of the printed films. Therefore, the conductivity *σ* of the films only changes by less than 10% after several bending cycles. We do not expect the other TE parameters *S* and *κ* to change with bending cycles as the crystallographic structure remains unchanged. Hence, the TE performance of the films will not be affected significantly after bending several times.

**Figure 4:**
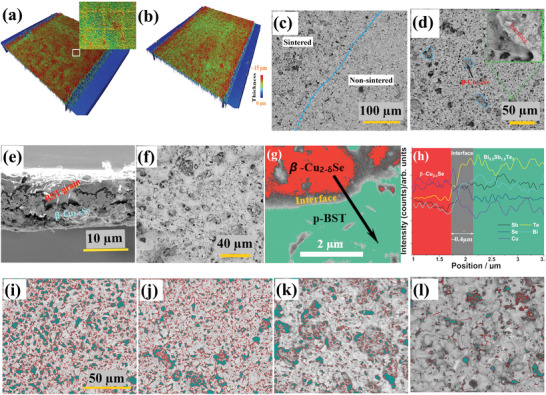
Morphology of the printed a) sintered film with *x* = 0.05 for the p‐BST and b) sintered film with *x* = 0.10 for *n*‐BT using WLI 3D microscope. Secondary electron microscopic (SEM) images of c–e) the sintered p‐BST film with *x* = 0.05 and f) the sintered n‐BT film with *x* = 0.10 were acquired at 5 kV. The image of the sintered p‐ and n‐type films signify two types of phase contrast (dark and bright gray regions). The cross‐section SEM image at higher magnification shows a close‐up of the two phases. g) A higher magnified phase contrast image of p‐BST film for *x* = 0.05 where line scan is performed and h) arrow indicates the location of the EDXS line scan from the *β* ‐Cu_2−_ *_*δ*_* Se to p‐BST phase. The surface morphology of the p‐BST films for *x* = i) 0, j) 0.05, k) 0.10, and l) 1 reveal the porous (dark cyan region with red border) nature of the films. The porosity (dark cyan region) in the film reduces with increasing IB.

The morphological and microstructural properties of the printed films influence the mechanical flexibility and the microscopic transport behavior across the grains. The film surfaces for all *x* are homogeneous and smooth at the macroscopic scale. The morphologies of the printed films recorded using the white light interferometer indicate homogeneous thicknesses across the surface (see **Figure** **4**a,b). The shallow micropores were created in the sintered films due to the expulsion of the organic ingredients during the photonic curing. The thickness of the printed films shrank from >20 to 10–15 µm as a result of the compaction of the grains after photonic curing. The microstructures of the printed p‐ and n‐type films for *x* > 0 indicate the formation of two distinct phases after sintering (see Figure 4c–f). The p‐BST film surface for *x* = 0.05 in the nonsintered region shows homogeneous shading, whereas phase contrast is appeared in the sintered area, indicating the formation of *β* ‐Cu_2−_ *_*δ*_* Se after sintering (see Figure 4c). The grain size in the nonsintered film varies from the nanoscale to several micrometers and fuses together after the sintering (see Figure S2, Supporting Information). The inset of Figure 4d shows the interface between a p‐BST grain and the *β* ‐Cu_2−_ *_*δ*_* Se. The lamellar microstructures are visible in the p‐BST grain region,^[^ ^40^ ^]^ where a clay‐like morphology is observed in the highly conducting *β* ‐Cu_2−_ *_*δ*_* Se region. A similar microstructure of *β* ‐Cu_2−_ *_*δ*_* Se film form by reacting Se and Cu through photonic sintering has been reported in the previous work.^[^ ^41^ ^]^ The elemental analysis of the p‐ and n‐type film confirms the formation of the two phases after sintering (c.f., Figure S2c,f, Supporting Information). The sintered flexible p‐BST film with *x* = 0.05 was cut, and its cross‐section secondary electron microscopic (SEM) image was recorded, showing two separate phases (see Figure 4e). There is also a possibility of leftover organic ingredients in the film. However, the residual decomposed polyvinylpyrrolidone (PVP) for *x* > 0 content would be much less than 1 wt% and it is very difficult to do a quantitative analysis.

A line scan across the interface between the p‐BST (green region) and *β*‐Cu_2−_ *_*δ*_* Se phase (red region) has been performed to detect the change in elemental composition (see Figure 4g,h). It is found that the at% of Cu and Se elements decrease and the at% of the Sb, Bi, and Te increase. The presence of the Cu and Se elements is observed at the interface (gray region) indicating the excess elements seem to be diffused into the p‐BST grain altering the crystal structure. From the XRD it is obvious that “*δ*” at% of excess Cu and “*δ*/2” at% of excess Se are supposed to be present in the IB lattice which probably diffuses into the TE phases. The Cu ions generally tend to intercalate into the Sb/Bi‐Te crystallite. They are prone to diffuse into the Sb/Bi‐Te grain parallel to cleavage planes with a diffusion coefficient (*D*) of ≈10^−5^ cm^2^ s^−1^ at elevated temperatures. The diffusion coefficient perpendicular to the cleavage planes is ≈10^−7^ cm^2^ s^−1^.^[^ ^42^ ^]^ The diffusion coefficient of Se into Bi/Sb‐Te is of the order of 10^−11^ cm^2^ s^−1^.^[^ ^43^ ^]^ Hence, the diffusion lengths of the Cu and Se into the Sb/Bi‐Te crystal can be estimated via $L_{D}=\sqrt{D\tau }\;$considering *τ* is the flash sintering time ≈8 × 10^−3^ s. The *L*_D_ is estimated to be a few micrometers in the van der Waals gap and less than a micrometer into the Bi/Sb‐Te platelets perpendicular to the cleavage planes for Cu, whereas the *L*_D_ is of the order of only nanometers for Se. Therefore, although the temperature reaches 800 K, the Cu/Se might not be able to fully diffuse across the large Bi/Sb‐Te grains during the ultrafast sintering process. It is also visible in the Energy‐dispersive X‐ray spectroscopy (EDXS) result that the TE grains are not surrounded by the Cu/Se uniformly. The Cu/Se is mostly concentrated outside the Bi/Sb‐Te grains and the Cu/Se concentration probably exponentially decreases inside the grains. Hence, the high conducting *β*‐Cu_2−_ *_*δ*_* Se phase outside of the TE grain facilitates modulation doping and improves the resulting electrical conductivity. However, it is very difficult to trace the elements clearly due to the presence of organic ingredients and porosities. It should be noted that the EDXS line scan only specifies the presence of the elements, not the exact composition. As we have performed a line‐scan in the backscattering mode, the signal from the deeper surface could influence the quantification of at% of an elemental.

The area fraction of the micropores present in p‐BST films for *x* = 0, 0.05, 0.10, and 1 have been analyzed using SEM images. It is found that the porosity in the films (the dark cyan region with a red border) decreases with increasing IB amount (see Figure 4i–l). The area fraction of the porous regions in the films estimated to be 35%, 27%, 20%, and 5% for *x* = 0, 0.05, 0.10, and 1, respectively. Although the estimations might not be matched with the actual volume fraction of the porous regions in the films, the results indicate that the addition of the IB improves the mechanical properties of the films. Therefore, the mechanical flexibility of the pristine films is found to be poor and it improves with the addition of IB. In addition, the charge and phonon transport properties of the films are affected by the micropores.

### 3.2 Thermoelectric Performance of the p‐ and n‐Type Printed Films

**Figure 5:**
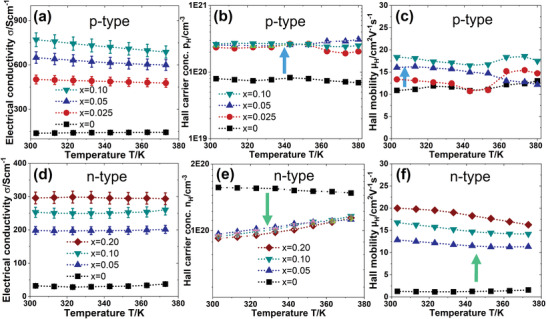
The variation of a) electrical conductivity (*σ*), b) Hall carrier concentration (*p*_H_), and c) Hall carrier mobility (*µ*_H_) of the printed p‐BST films for *x* = 0–0.1 with temperature after sintering. The temperature‐dependent d) electrical conductivity (*σ*), e) Hall carrier concentration (*n*_H_), and f) Hall carrier mobility (*µ*_H_) of the printed n‐BT films for *x* = 0–0.2.

The temperature‐dependent electronic transport properties of the p‐ and n‐type sintered films were studied for 0 ≤ *x* ≤ 0.2 from 300 to 400 K (see **Figure** **5**). The RT transport parameters are given in **Table** **1** for all the films. The Hall carrier concentration *p*_H_ of the p‐BST films increases from 0.79 × 10^20^ cm^−3^ for *x* = 0 to 2.62 × 10^21^ cm^−3^ for *x* = 0.10 where the carrier concentration of the n‐BT film *n*_H_ decreases from 1.57 × 10^20^ cm^−3^ for *x* = 0 to 0.94 × 10^20^ cm^−3^ for *x* = 0.10 due to an increase of the *β*‐Cu_2−_ *_*δ*_* Se phase in the printed films. The pure *β*‐Cu_2−_ *_*δ*_* Se‐based film (*x* = 1) is a highly doped p‐type material, and its *p*_H_ is found to be two orders of magnitude higher (≈8 × 10^21^ cm^−3^) than pristine p‐BST/n‐BT film.^[^ ^41^ ^]^ The intercalation of the Cu in the van der Waals gap generally exhibits a donor‐like behavior. In contrast, IB shows an acceptor‐like behavior for both the p‐ and n‐type films, indicating a dominant influence of *β*‐Cu_2−_ *_*δ*_* Se over intercalated Cu. Hence, the resulting *p*_H_ increases in the p‐BST films, whereas *n*_H_ decreases in the n‐BT films due to the addition of the IB. An analogous effect has been reported; incorporating p‐type Cu_2_Te in the n‐type Bi_2_Te_2.7_Se_0.3_ reduces the total carrier concentration behaving like an acceptor doping.^[^ ^44^ ^]^ Similarly, the carrier concentration is decreased in n‐type Bi_2_Te_3_ and increased in the p‐type Bi_2_Te_3_ by incorporating the p‐type poly(3,4‐ethylenedioxythiophene) polystyrene sulfonate (PEDOT:PSS).^[^ ^45^ ^]^

**Table 1:** The RT transport parameters, charge carriers concentration (*p*_H_), Hall carrier mobility (*µ*_H_), and Hall coefficient (*R*_H_) of sintered printed p‐ and n‐type films

Films	Electrical conductivity *σ* [S cm^−1^]		Hall mobility *µ*_H_ [cm^2^ V^−1^ s^−1^]		Hall carrier concentration *n*_H,_/*p*_H_ [×10^20^ cm^−3^]		Hall coefficient *R*_H_ [×10^−2^ cm^3^ C^−1^]	
	*P*	*n*	*p*	*n*	*p*	*n*	*p*	*n*
*x* = 0	139	32	10.9	1.1	0.79	1.57	7.8	−3.9
*x* = 0.025	501	—	13.3	—	2.34	—	2.7	—
*x* = 0.05	645	198	13.4	12.8	2.53	0.96	2.0	−6.5
*x* = 0.10	770	253	18.3	16.8	2.62	0.94	2.4	−6.6
*x* = 20	—	296	—	20	—	0.92	—	−6.8

The Hall mobility *µ*_H_, increases by 50% in the p‐BST film from 11 to 18 cm^2^ V^−1^ s^−1,^ while that increases by 15 times in the n‐BT film for *x* = 0.10. As a result, the electrical conductivity *σ* of the p‐BST film increases from 139 to 770 S cm^−1^ when *x* increases from 0 to 0.1 because of the increase in both *p*_H_ and *µ*_H_. The *µ*_H_, slightly decreases with increasing temperature in the p‐BST films for *x* > 0 due to the more disordered Cu‐ions. On the other hand, although the *n*_H_ of the n‐BT decreases by ≈40% for *x* > 0, a considerable enhancement in *µ*_H_, increases the overall *σ*. The bulk p‐BST/n‐BT alloy generally shows higher mobility >100 cm^2^ V^−1^ s^−1^.^[^ ^41^ ^]^ However, the organic ingredients between p‐BST/n‐BT grains in the pristine film increase the interfacial resistance suppressing the carrier mobility. The formation of high conducting paths of *β*‐Cu_2−_ *_*δ*_* Se across the p‐BST/n‐BT grains reduces the interfacial resistance, improving the mobility in the composite material for *x* > 0. The resultant *σ* is found to decrease with increasing temperature, a behavior of a degenerate semiconductor. Similar transport phenomena were observed in the (BiSb)_2_Te_3_‐based and *β*‐Cu_2−_ *_*δ*_* Se‐based TE materials. Consistent with 3D continuum models, the percolation threshold for a two‐phase system with spherical particles is ≈29 vol% of any of the two phases.^[^ ^46^, ^47^ ^]^

**Figure 6:**
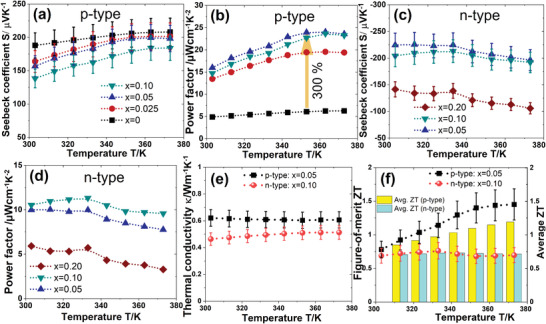
The temperature‐dependent TE parameters a) Seebeck coefficient *S* and b) power factor *S* ^2^ *σ* of the sintered p‐BST films. The temperature‐dependent c) *S* and d) power factor *S* ^2^ *σ* of the sintered n‐BT films. The TE film with *x* = 0.05 for p‐BST and *x* = 0.10 for n‐BT exhibit the highest power factor at all the temperatures. e) The variation of the thermal conductivity and f) the figure‐of‐merit ZT and average ZT of the p‐BST for *x* = 0.05 and n‐BT films for *x* = 0.10.

Therefore, the TE properties of the films are dominated by the p‐BST/n‐BT phase. The pristine p‐type film shows the highest RT positive Seebeck coefficients S of 188 µV K^−1^, whereas the *S* for the pristine n‐BT film was not possible to be determined due to the creation of the cracks after sintering in the films. The Seebeck coefficient *S* decreases with increasing *x* for both p‐BST and n‐BT films (see **Figure** **6**a,c). The *S* is positive for all the p‐BST films and negative for n‐BT films with *x* > 0, confirming the p‐ and n‐type nature of the films, respectively. S increases for the p‐BST films and decreases for the n‐BT films with increasing temperature. The temperature‐dependent TE power factor (*S* ^2^ *σ*) of the p‐ and n‐type films were estimated from *S* and *σ* and shown in Figure 6b,d.

The power factor of the photonic‐sintered p‐BST film improves by ≈220% at RT and by ≈300% at 350 K due to the addition of the secondary phase *β*‐Cu_2−_ *_*δ*_* Se, and it increases with temperature for all compositions. The sintered p‐BST film with *x* = 0.05 and n‐BT film with *x* = 0.10 exhibit the highest power factor values. The optimized p‐BST film shows a power factor of 16 µW cm^−1^ K^−2^ at RT and 24 µW cm^−1^ K^−2^ at 350 K. The value is ten times higher than the pristine p‐BST film synthesized by the conventional vacuum sintering process. The n‐BT film shows a power factor of 10.5 µW cm^−1^ K^−2^ at ≤330 K. The power factor values are notably higher than reported pressure treatment‐free TE printed films. The *V*p and composition‐dependent power factor on glass and flexible substrates is discussed in the Supporting Information (see Figure S3, Supporting Information). The in‐plane thermal conductivity *κ* of p‐ and n‐type sintered films on a thin film analyzer (TFA) chip is estimated by 3*ω* method. The TFA chip is built on a silicon wafer where two thin heaters with a width <5 µm are deposited on free‐standing Si3N4 membranes of 300 nm surrounded by an Au rim. The heaters are connected in a four‐wire configuration. The *κ* for the pristine printed film is found to be ≈0.40 W m^−1^ K^−1^. The *κ* for both p‐ and n‐type films increases with increasing *x* and it does not change significantly with temperature. The *κ* is found to be 0.62 W m^−1^ K^−1^ for p‐BST film with *x* = 0.05 and 0.47 W m^−1^ K^−1^ for n‐BT film with *x* = 0.10 at RT. The overall estimated *κ* values of the printed films are lower than their bulk counterparts due to low density (<50% of theoretical density) and higher interfacial resistance caused by micropores and leftover organic ingredients. Assuming p‐BST particles are spherical in the pristine printed p‐BST film, the low thermal conductivity of the film can be explained using the Bruggeman model. The thermal conductivity *κ* of a film containing p‐BST particles and pores can be expressed as^[^ ^30^ ^]^

(5) $$\left(1-\varphi_{p-\mathrm{BST}}\right)\;=\frac{\left(\kappa -\kappa_{\mathrm{compact}}\right)}{\left(\kappa_{\mathrm{Air}}-\kappa_{\mathrm{compact}}\right)}\;{\left(\frac{\kappa_{\mathrm{Air}}}{\kappa }\right)}^{1/3}$$

where *κ* and *κ*_compact_ are the thermal conductivities of the printed film and the compacted p‐BST film, respectively, *φ*_p − BST_ is the volume fraction of the p‐BST particles and *κ*_Air_ is the thermal conductivity of the air. The volume fraction of the PVP and porosity in the film are estimated to be ≈12% and 35%, respectively. Hence, for simplicity, the *κ* is calculated using *φ*_p − BST_ ≈ 0.53 and the *κ*_Air_ ≈ 0.025 W m^−1^ K^−1^. The resultant *κ* of the pristine printed P‐BST film is estimated to be 0.48 W m^−1^ K^−1^ which is consistent with the measured value of 0.40 W m^−1^ K^−1^_._ The total *κ* comprises lattice thermal conductivity (*κ*_l_) and electronic thermal conductivity (*κ*_el_), they were determined using Wiedmann‐Franz Law (*κ*_el_ = *σLT*, with *L* the Lorentz number) and the single parabolic model. A more detailed discussion on the *κ*_l_ and *κ*_el_, is given in Figure S4 in the Supporting Information. The figure‐of‐merits ZT of the films has been calculated from their power factor values and thermal conductivities. The p‐BST film with *x* = 0.05 exhibits the highest ZT at all temperatures. A ZT of 0.8 at RT and 1.45 at 373 K are achieved in the p‐BST film for *x* = 0.05. The RT ZT is found to be ≈0.75 for *x* = 0.10 in the n‐BT film and it decreases at high temperature. The ZT values are comparable to their bulk counterparts. The average ZT has been calculated for both films, an important parameter for device applications. Both p‐ and n‐type films in the temperature range 300–380 K exhibit high average ZT values. To the best of our knowledge, these pair of ZT values are the highest ZTs achieved in pressure treatment‐free printed p‐ and n‐type TE films. The TE films are found to be stable, reproducible and yield a similar RT power factor of 15 µW cm^−1^ K^−2^ (see Figure S3, Supporting Information).

### 3.3 Fabrication and Performance of the Folded TEGs (f‐TEGs)

#### 3.3.1 Fabrication of the f‐TEGs

**Figure 7:**
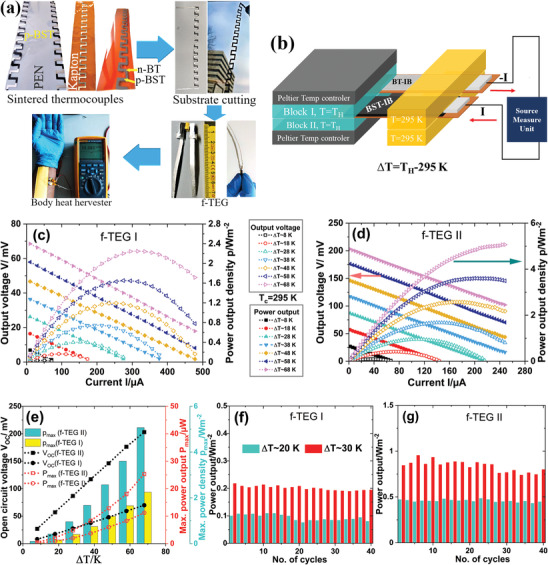
a) Step‐by‐step fabrication process of the f‐TEGs; printing‐sintering‐creasing‐folding and the f‐TEG as body heat harvester. b) The schematic diagram of the TEG characterization setup. The current‐dependent output voltage and output power of c) the single‐legged and d) double‐legged f‐TEGs for different ∆*T*s. The change in the open‐circuit voltage (*V*_OC_), maximum power output (*P*_max_), and maximum power density (*p*_max_) of the print‐TEG with ∆*T*s (e). The stability and the repeatability of the performance in f) the single‐legged and g) double‐legged f‐TEGs after running several temperature cycles.

The routine of the fabrication process of the folded f‐TEGs is shown in **Figure** **7**a. We have used the optimized p‐BST film with *x* = 0.05 and n‐BT film with *x* = 0.10 for the device fabrication on PEN and Kapton. Although the n‐BT films with *x* = 0.05 and 0.10 show similar ZT, the film with *x* = 0.10 was used for device applications to minimize the device contact resistance. At first, two single‐legged f‐TEGs (f‐TEG I) were fabricated using p‐BST film with *x* = 0.05 and 0.10 on PEN and one single‐legged f‐TEG I was fabricated on Kapton using p‐BST film with *x* = 0.05. For simplicity, we have used printed silver legs to complete the thermocouple. An array of 13 Z‐shaped silver patterns (3 mm × 12 mm × 0.005 mm; shown in white in Figure 7a was printed on the flexible substrates (PEN and Kapton). The silver pattern was dried at 373 K for 5 min. A mirror of the Z‐shaped array was printed using the p‐BST‐IB TE ink for *x* = 0.05 and 0.10 with an individual TE leg dimension of 3 mm × 12 mm × 0.01 mm to fabricate a square wave‐like structure (shown in dark gray in Figure 7a). The 13 TE legs were connected in series by overlapping the silver and p‐BST‐IB legs. The final printed pattern was then dried at 343 K for 5–10 min on a hot plate, then sintered using a photonic‐curing process. The nonprinted area of the substrates was cut out after sintering the printed thermocouples (Video S1, Supporting Information). Then, the thermocouples were creased at the junctions using a hot blade. Finally, it was folded at the creased positions to fabricate PEN‐ and Kapton‐based *π*‐shaped TEGs (f‐TEGs) (Video S2, Supporting Information). Later, the silver legs were replaced by the n‐BT film with *x* = 0.10 to fabricate a p‐BST and n‐BT based double‐legged f‐TEG II. In this case, an extra layer of silver was printed at the overlap region to avoid discontinuity during folding. The dimensions of the resultant PEN‐ and Kapton‐based f‐TEGs were ≈10 mm × 12 mm × 0.8 mm and 10 mm × 12 mm × 0.5 mm, respectively. The effective heat flux area from the hot to the cold side was ≈8 mm^[^ ^2^ ^]^ for PEN and 5 mm^[^ ^2^ ^]^ for Kapton substrate.

#### 3.3.2 The Performance of the f‐TEGs

The performances of the p‐BST based single‐legged f‐TEG I and p‐BST:n‐BT based double‐legged f‐TEG II are shown in Figure 7c–g and Figure S6 in the Supporting Information. The f‐TEGs were characterized by the maximum power point tracking method varying the device current from −600 to 600 µA and the temperature difference (Δ*T*) between the hot and cold sides from 8 to 68 K. One side of the f‐TEGs is kept at 295 K and the other side was clamped by two metal blocks. The Δ*T* was varied from 8 to 68 K by changing the temperature of the metal blocks from 303 to 363 K (see Figure 7c). For the p‐BST‐based single‐legged f‐TEG I with *x* = 0.05, a similar power output *P* is realized in both the PEN‐ and Kapton‐based f‐TEGs.

An open‐circuit voltage *V*_OC_ of ≈70 mV with a maximum power output *P*_max_ of ≈11 µW is exhibited by the singled PEN‐ and Kapton‐based f‐TEGs for Δ*T* = 68 K (Figure 7c and Figure S6, Supporting Information). The *V*_OC_ and *P*_max_ increase with increasing the Δ*T*, mainly because of the higher heat flux through the f‐TEGs for a higher temperature gradient where the overall heat transfer coefficient is considered to be unchanged. However, a higher maximum power density *p*_max_ of ≈2.2 W m^−2^ is achieved in the Kapton‐based f‐TEG I as compared to the 1.4 W m^−2^ in the PEN‐based f‐TEG I for Δ*T* = 68 K. Lower thickness of the Kapton substrate (25 µm) enhances the overall power density of the Kapton‐based single‐legged f‐TEG I. Another PEN‐based f‐TEG I with *x* = 0.10 was fabricated for comparison and characterized for Δ*T* = 8–48 K (see Figure S6, Supporting Information). The performance of the PEN‐based f‐TEGs with *x* = 0.05 and 0.10 is compared in Figure S6d in the Supporting Information. The output power for *x* = 0.05 is higher for all Δ*T*s, which is consistent with our findings for the power factor and signifies possession of a higher thermal conductivity (see Figure 6). The maximum power output density *p*_max_ increases by more than two times in the p‐BST and n‐BT based double‐legged f‐TEG II on Kapton. A *p*_max_ of ≈5.1 W m^−2^ with a *V*_OC_ of 203 mV is achieved in the Kapton‐based double‐legged f‐TEG II for Δ*T* = 68 K (see Figure 7d). Although the average ZT of the n‐BT (*x* = 0.10) film is lower than that of the p‐BST (*x* = 0.05) film, the reduction of the parasitic heat flow through the Ag films helps double the power output of the double‐legged f‐TEG II. The stability and repeatability of the single‐ and double‐legged f‐TEGs on Kapton have been studied after a few days of their characterization by running them again several cycles for two mid‐range Δ*T*s 20 and 30 K (see Figure 7f,g). The single‐ and double‐legged f‐TEGs yield similar maximum power density for 40 cycles. The standard deviations vary between 1% and 3% from the mean value of the *p*_max_ for the f‐TEGs for ∆*T* = 20 and 30 K. An output voltage of 11.3 mV is executed when the ‐TEG is attached to the body skin. The estimated Seebeck coefficient per leg for all the f‐TEGs is lower than the material Seebeck coefficient. In general, the thermal contact resistance, parasitic heat flow through the substrates and Ag connectors, and Joule heating significantly reduce the thermal output voltage. Because of this, the actual temperature difference between the hot and cold ends of the TE films is lower than its set value *∆T*. Hence, lower thermal voltage is generated, and a lower Seebeck coefficient is estimated.

### 3.4 Discussion

The primary focus of printed TE materials has been on good printability with bulk‐like TE performance. In recent years, a significant advance has been made to improve printability and performance.

**Figure 8:**
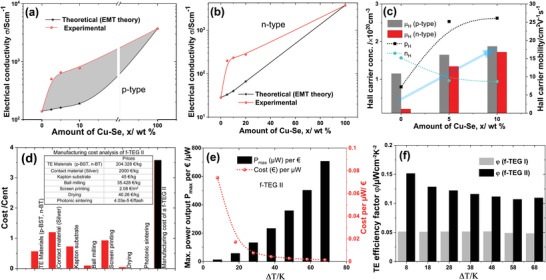
Comparison of theoretical and experimental composition‐dependent electrical conductivity *σ* for a) the p‐BST and b) n‐BT films. c) Variation of the charge carrier concentration and mobility with *x* for both the films. d) Manufacturing cost analysis of an f‐TEG II. e) The maximum power output *P*_max_ per € and the cost to produce 1 µw power with ∆*T*. f) The variation of the TE efficiency factor *φ* of the f‐TEG I and f‐TEG II with ∆*T*.

However, to meet the objective of the printed thermoelectrics, the printed TE materials also require mechanical flexibility and robustness to withstand manufacturing processes. Furthermore, the manufacturing process should be controllable and fast. In this work, we have overcome three major bottlenecks in printed thermoelectrics: a) printability b) high TE performance, and c) mechanical flexibility. In addition, the manufacturing process is reproducible and identical for both p‐BST and n‐BT films. Hence, one‐pot ink formulation, screen printability, and rapid photonic curing can be upscaled toward a roll‐to‐roll mass‐scale production of TEGs. Here, the high conducting *β*‐Cu_2−_ *_*δ*_* Se phase is grown at the interfaces through fast photonic curing reducing the interfacial resistance in the printed p‐BST and n‐BT films. Hence, the “microsoldering” of p‐BST/n‐BT grains with the highly conducting *β*‐Cu_2−_ *_*δ*_* Se enhances charge carrier transport across the grain boundaries minimizing the detrimental effects of leftover organic ingredients. Consequently, the *µ*_H_ increases with increasing *x* for both p‐ and n‐type films, enhancing their *σ*. The highly doped p‐type nature of the *β*‐Cu_2−_ *_*δ*_* Se phase increases the *p*_H_ in p‐BST films, whereas it decreases the *n*_H_ in the n‐BT films (see **Figure** **8**c). The increase in the *µ*_*H*_ is more prominent than the decrease in the *n*_H_ in the n‐BT films; hence, the resultant *σ* increases significantly for *x* > 0. The experimental composition‐dependence is compared with the theoretically calculated value predicted by effective medium theory (c.f., Figure 8a,b). The experimental values are found to be higher compared to the theoretical value for a particular *x*. This signifies that the formation of the *β*‐Cu_2−_ *_*δ*_* Se phase not only increases the overall *σ* due to its high conductivity but are also modifying grain interfaces, increasing the *µ*_H_ of the films.

The f‐TEGs fabricated using the p‐type BST and n‐type BT printed TE materials exhibit a promising power density up to ≈5.1 W m^−2^. A comparison of the materials' power factor and maximum power outputs per thermocouple of the printed TEGs fabricated using the same materials is given in Figure 1b. The performance of the f‐TEGs is found to be superior, along with their mechanical robustness. The manufacturing cost of the f‐TEG II is estimated to be ≈3.58 cent per piece (see Figure 8d). The *P*_max_ per euro increases with increasing Δ*T*s reaching 710 µW € for Δ*T* = 68 K. Hence, the cost to produce 1 µW power is 0.0014 € for Δ*T* = 68 K. However, the cost can potentially be reduced further by reducing contract resistance and by increasing the number of printed TE elements per area. The total power output of a double‐legged f‐TEG II made of printed p‐BST and n‐BT printed materials is proportional to its internal resistance *R*_I_, the total number of thermocouples (*N*), and the temperature difference *∆T* between the hot and cold sides. Hence, the f‐TEG II power output can be expressed as

(6) $$P\;=\frac{V_{\mathrm{OC}}^{2}}{{\left(R_{L}+R_{I}\right)}^{2}}\;R_{L}=\frac{{\left[\left(S_{\mathrm{BST}}-S_{\mathrm{BT}}\right)\times \left(\Delta T\times N\right)\right]}^{2}R_{L}}{{\left(R_{L}+R_{I}\right)}^{2}}\;$$

where *R*_I_ is the internal device resistance ( *R*_I_ = *R*_contacts_ + *R*_films_), *R*_L_is the load resistance, *S*_BST_ and *S*_BT_ are the Seebeck coefficients of the printed p‐BST and n‐BT films, respectively. The *R_I_* for the TEG I and TEG II were 128 and 362 Ω respectively. *P*_max_ is achieved for *R*_I_ = *R*_L_ . The power output of the double‐legged f‐TEG II could be enhanced by increasing the leg dimension and by the leg number. Hence, a 1 cm thick f‐TEG II with an active area of ≈2 cm^2^ could deliver a power output of ≈100 µW for a ∆*T* = 28 K. The power output of an f‐TEG II with a cost of <1 € is sufficient to power different IoT devices. The TE efficiency factor ($\varphi =p_{\max}/\Delta T^{2}$) of the f‐TEGs are calculated (see Figure 8f).^[^ ^48^ ^]^ The f‐TEG II exhibits a *φ* of 0.152 µW cm^−2^ K^−2^ for ∆*T* = 8 K, which is higher than most of the reported values for thick and thin film TEGs.^[^ ^48^ ^]^ However, the *φ* of the f‐TEG II is not up to the expectation as the ZT values of the p‐ and n‐type legs are high. Apart from the average ZT, three major factors govern the power density/the *φ* of the f‐TEGs: a) Fill factor, b) thermal contact resistance, and c) electrical contact resistance of the TEG. They have been discussed below.

#### 3.4.1 Fill Factor

The fill factor of the f‐TEGs is estimated to be only ≈15.6%. Even if it is considered that the f‐TEGs were perfectly thermally contacted during measurement, the heat flux through only 15.6% of the total device cross‐sectional area is converted to electric power. However, the fill factor can be enhanced by increasing the thickness‐ratio of the TE leg to the substrate and by decreasing the distance between the n‐ and p‐ legs of the f‐TEGs. It is important to mention that an increase in the leg thickness causes the deterioration of the mechanical flexibility of the TE legs. Nevertheless, although the fill factor of the f‐TEGs is low, it successfully yields sufficient power to run IoT devices. Hence, low‐cost f‐TEGs could be employed for energy harvesting applications, while its fill factor needs to be enhanced for waste heat recovery applications.

#### 3.4.2 Thermal Contact Resistance

For a TE device, the thermal resistance between the device and the source or sink obstructs the heat flow, reducing the device's efficiency. For the same reason, the actual Δ*T* is found to be lower than the set value of the Δ*T*, which leads to the underestimation of the thermovoltage. Due to the presence of insulating substrate and air inside the folded substrate, heat flow between the clamped blocks to the f‐TEGs might be obstructed. Hence, proper thermal contact and a suitable heat sink could enhance the power output density.

#### 3.4.3 Electrical Contact Resistance

The fraction of total device resistance (*R*_I_ in the range of 30–40% is contributed by the contact resistance (*R*_contacts_). Although the used conducting silver ink offers excellent flexibility and high conductivity, the contact resistance between the printed silver and the TE legs is significantly high. Hence, the high contact resistance diminishes the power output of the f‐TEGs. Other metal inks, including Cu could also work better. In addition, a printed intermediate diffusion layer (a layer between the TE film and the interconnecting materials) could be employed to reduce the contact resistance.

## 4 Conclusion

It is a challenge to prevail over the entanglement of good printability, high performance, and flexibility in printed thermoelectrics. All three qualities are required to achieve the goal of printed thermoelectrics. In this work, we have employed photonic curing technology to sinter pairs of p‐BST and n‐BT‐based films on flexible substrates containing a Cu‐Se‐based inorganic binder. Ball milling of the materials enables excellent printability, while the photonic curing of (p‐BST/n‐BT)‐IB printed films results in outstanding mechanical flexibility. The superior thermoelectric behavior can be traced down to the inorganic binder, which reduces the interfacial resistance by forming a high *σ β*‐Cu_2−_ *_*δ*_* Se through sintering. We have achieved: a) printability, b) high TE performance, and c) mechanical flexibility in a pair of p‐ and type printed films. The p‐type TE film with a maximum ZT of ≈0.1.45 and the n‐type TE film with a ZT of ≈0.75 are employed to fabricate fully printed f‐TEGs. The double‐legged f‐TEG II demonstrated a high power density of 5.1 W m^−2^ for ∆*T* = 68 K.

## 5 Experimental Section

### Materials

Ingots of p‐type Bi_0.5_Sb_1.5_Te_3_ and n‐type Bi_2_Te_2.7_Se_0.3_ (beads, 99.99% trace metals basis, Sigma‐Aldrich), Se powder (100 mesh, ≥99.5% trace metals basis, Sigma‐Aldrich), copper powder (spheroidal) (10–25 µm, 98%, Sigma‐Aldrich), PVP (average *M*w ≈40 000, Sigma‐Aldrich), *N*‐methyl‐2‐pyrrolidone (NMP) (anhydrous, 99.5%, Sigma‐Aldrich), silver ink (LOCTITE ECI 1010 E&C), PEN (25 µm, DuPont de Nemours), and Kapton (25 µm, DuPont de Nemours) were used.

### Preparation of Printable Inks and Printed TE Films

**Figure 9:**
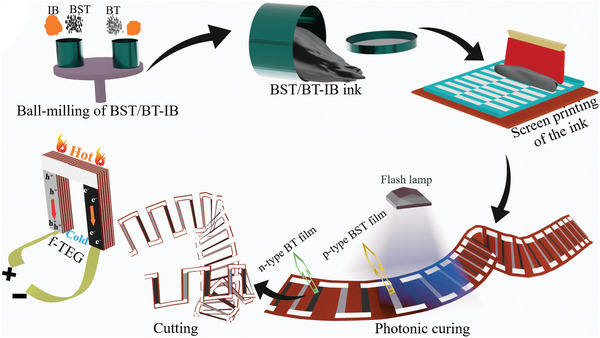
Illustration of the synthesis processes of the printable inks, p‐BST/n‐BT‐IB printed TE films and the f‐TEG fabrication.

First, Bi_0.5_Sb_1.5_Te_3_ (p‐BST) and Bi_2_Te_2.7_Se_0.3_ (n‐BT) were ground into micrometer‐sized powder using a mortar and pestle in the presence of N_2_. The obtained TE powder was blended with the IB comprised of Cu, Se, and NMP‐PVP solution (8:92 in wt ratio), wherein the molar ratio of the Cu powder to the Se powder was 2 :1. The p‐BST/n‐BT and Cu‐Se content (by weight) in the total amount of metal powder are expressed as [(1−*x*)BST/BT‐(*x*)IB], x is in the range of 0 < *x* ≤ 0.20. The weight ratio of the (1−*x*)BST/BT‐(*x*)IB to the NMP‐PVP was 4:1. The resultant blends were then put inside two zirconia jars of 120 mL (one for p‐type and another for n‐type material) containing 10 mm size zirconia balls. The weight ratio of the balls to the total amount of constituents was 10:1. Then, the jars were closed and subsequently purged with Ar for 10 min. The samples were then milled using a Fritsch Planetary Mill PULVERISETTE 5 premium line at 200 rpm for 45 min. The resultant inks were printed on glass substrates and flexible PEN, Kapton substrates using a semi‐automated ROKUPRINT screen‐printing machine with a screen specification of 600 × 300 90–40 y/22° Hitex. The printed films were dried at 343 K for 5–10 min. Finally, the dried printed films were sintered by the millisecond‐photonic‐curing process. The schematic diagram of the film preparation process is shown in **Figure** **9**.

### The Characterizations of the Printed Films and the Devices

The crystallographic structures and phase analysis of the printed films were done by the RT XRD technique on a Bruker D8 diffractometer. The diffractometer was equipped with a Lynxeye XE detector in Bragg–Brentano geometry using Ni filtered Cu *K*_*α*1,2_ lines. The temperature‐dependent transport properties of the printed films [(1 − *x*)BST/BT‐(*x*)IB] were studied using a Hall measurement setup (Linseis HCS 10). The transport parameters Hall coefficient (*R*_H_), carrier concentration (*p*_H_/*n*_H_), Hall mobility (*µ*_H_), and electrical conductivity (*σ*) were determined from RT to 400 K. The temperature‐dependent Seebeck coefficient (*S*) of the printed films was measured using a custom‐built setup. The working mechanism of the custom‐built setup was described in a previous report.^[^ ^9^ ^]^ The in‐plane thermal conductivity (*κ*) of the printed films was determined using a Linseis TFA system. The measurement setup was developed based on the method presented by Völklein et al.^[^ ^49^, ^50^ ^]^ (see Figure S5, Supporting Information). The relative measurement errors linked with *S*, *σ*, and *κ* are 10%, 6%, and 10%, respectively. The surface morphology and thicknesses of the printed films were determined using a Bruker 3D microscope based on white‐light interferometry (WLI). The elemental and the microstructural analyses were done in secondary electron and backscattered electron modes using an FEI Quanta 650 environmental scanning electron microscope equipped with an solid state detector (SSD) and a Schottky field emitter operated with 5 and 15 kV. The flexibility of the TE films was studied by measuring the change in their resistance with the bending cycles using a digital protractor. The performance of the folded TEGs (f‐TEGs) was analyzed using a maximum power point tracking method by a KEITHLEY Source Measuring Unit 2601B. The detailed working principle of the device characterization setup methods was described in a previous report.^[^ ^9^ ^]^

## Conflict of Interest

The authors declare no conflict of interest.

## Supporting information

Supporting Information

Supplemental Video 1

Supplemental Video 2

## Data Availability

The data that support the findings of this study are available from the corresponding author upon reasonable request.
