# Supplementary material for: High Figure‐of‐Merit Telluride‐Based Flexible Thermoelectric Films through Interfacial Modification via Millisecond Photonic‐Curing for Fully Printed Thermoelectric Generators
Source: Adv Sci (Weinh). 2022 Sep 14;9(31):2202411. doi: 10.1002/advs.202202411 (PMC9631075; doi:10.1002/advs.202202411)
Supplement: Supplementary file 1 — Supporting Information [file ADVS-9-2202411-s002.pdf]

Supporting Information

High ZT telluride-based flexible thermoelectric films through interfacial modification via millisecond photonic-curing for fully printed TEGs

Md Mofasser Mallick, Leonard Franke, Andres Georg Rösch, Holger Geßwein, Zhongmin Long, Yolita M. Eggeler and Uli Lemmer**

M.-M. Mallick, L. Franke, A.-G. Rösch, U. Lemmer

Light Technology Institute, Karlsruhe Institute of Technology, 76131 Karlsruhe, Germany.

Email: uli.lemmer@kit.edu; mofasser.mallick@kit.edu.

U. Lemmer

Institute of Microstructure Technology, Karlsruhe Institute of Technology, 76344 Eggenstein-Leopoldshafen, Germany

H. Geßwein

Institute for Applied Materials, Karlsruhe Institute of Technology, 76344 Eggenstein-Leopoldshafen, Germany

Z. Long, Y.-M. Eggeler

Laboratory for electron microscopy, Karlsruhe Institute of Technology, 76131 Karlsruhe, Germany.

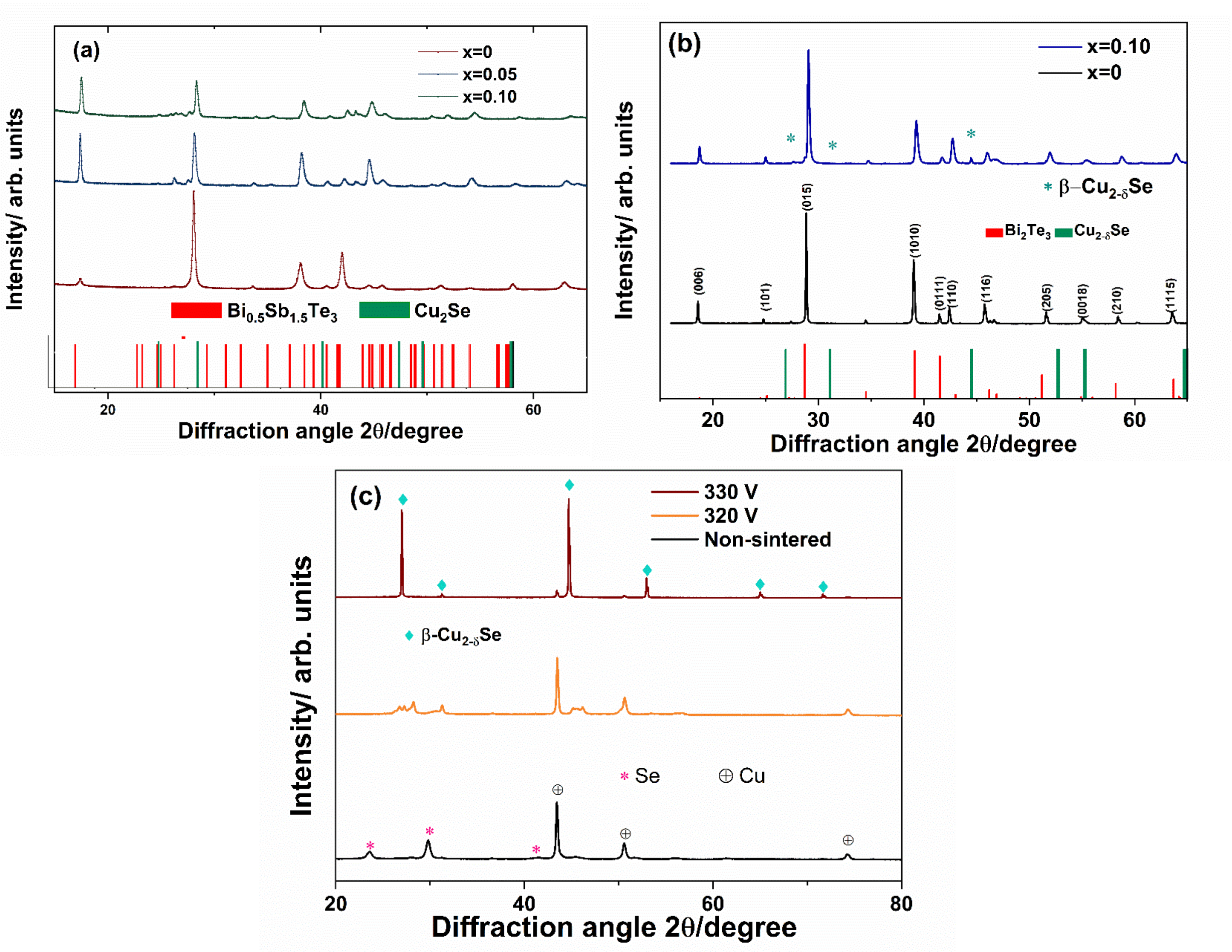

Figure S1: The XRD patterns of the p-BST (a) and n-BT (b) films at RT for 0≤x≤0.10. The XRD patterns of the non-sintered and sintered IB films (c). β-$Cu_{2-\delta}Se$ phase is found to form for Vp=330 V.

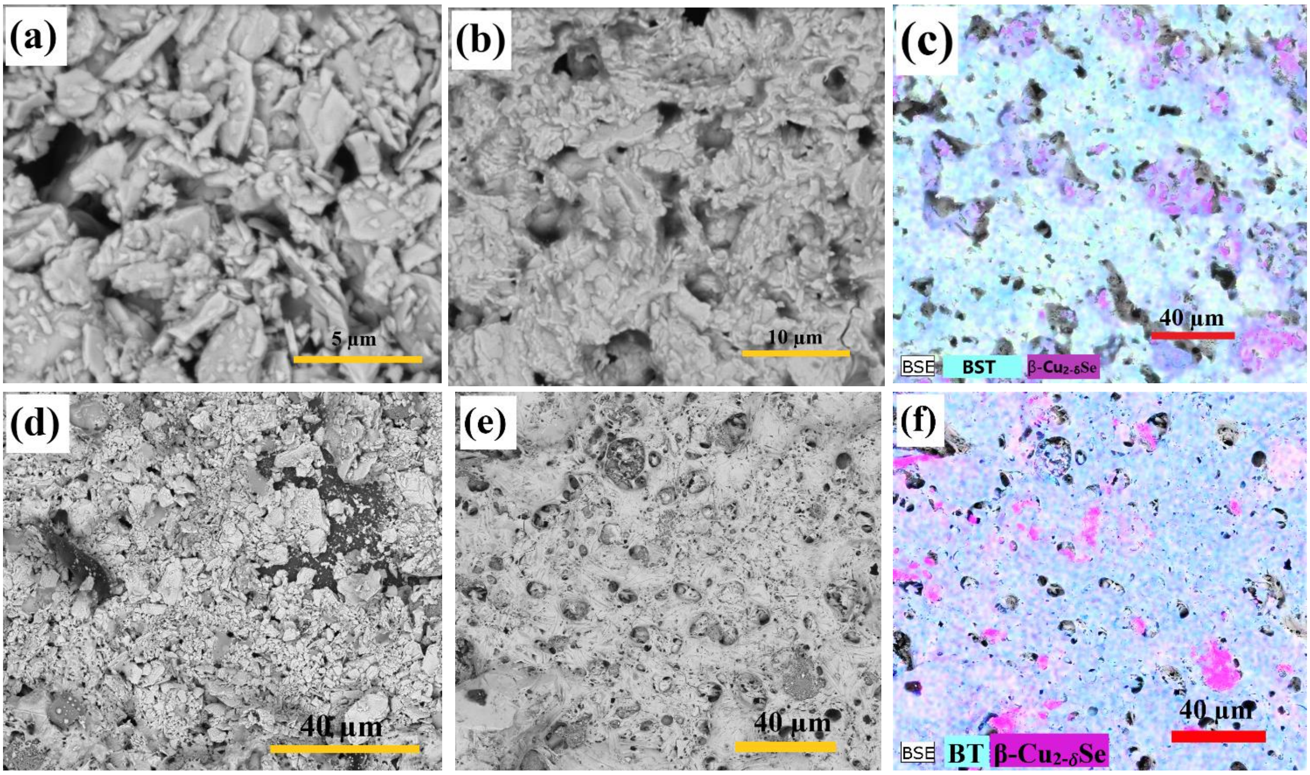

Figure S2: The SEM micrographs of the non-sintered (a) and sintered (b) p-BST films with x=0.05. The SEM images of the non-sintered (d) and sintered (e) n-BT films for x= 0.10. The elemental mapping of the p-BST (c) and n-BT (f) films. The pink regions indicate the β-$Cu_{2-\delta}Se$ phase. The EDS elemental mapping only indicates in a non-quantitative manner the presence of the elements. However, it is seen that the Cu-signal is strong in the pink regions while the Bi-, Sb-, and Te- signals show is pronounced in the bluish regions. Although the Se-signal is stronger in the pink regions, the noisy Se-signal also presents in the other regions. This is due to the volatile nature of the Se elements and the fact that we performed the elemental mapping in the backscattering mode. Thus, signals from the deeper layers of the films could superimpose the signals from the top surface.

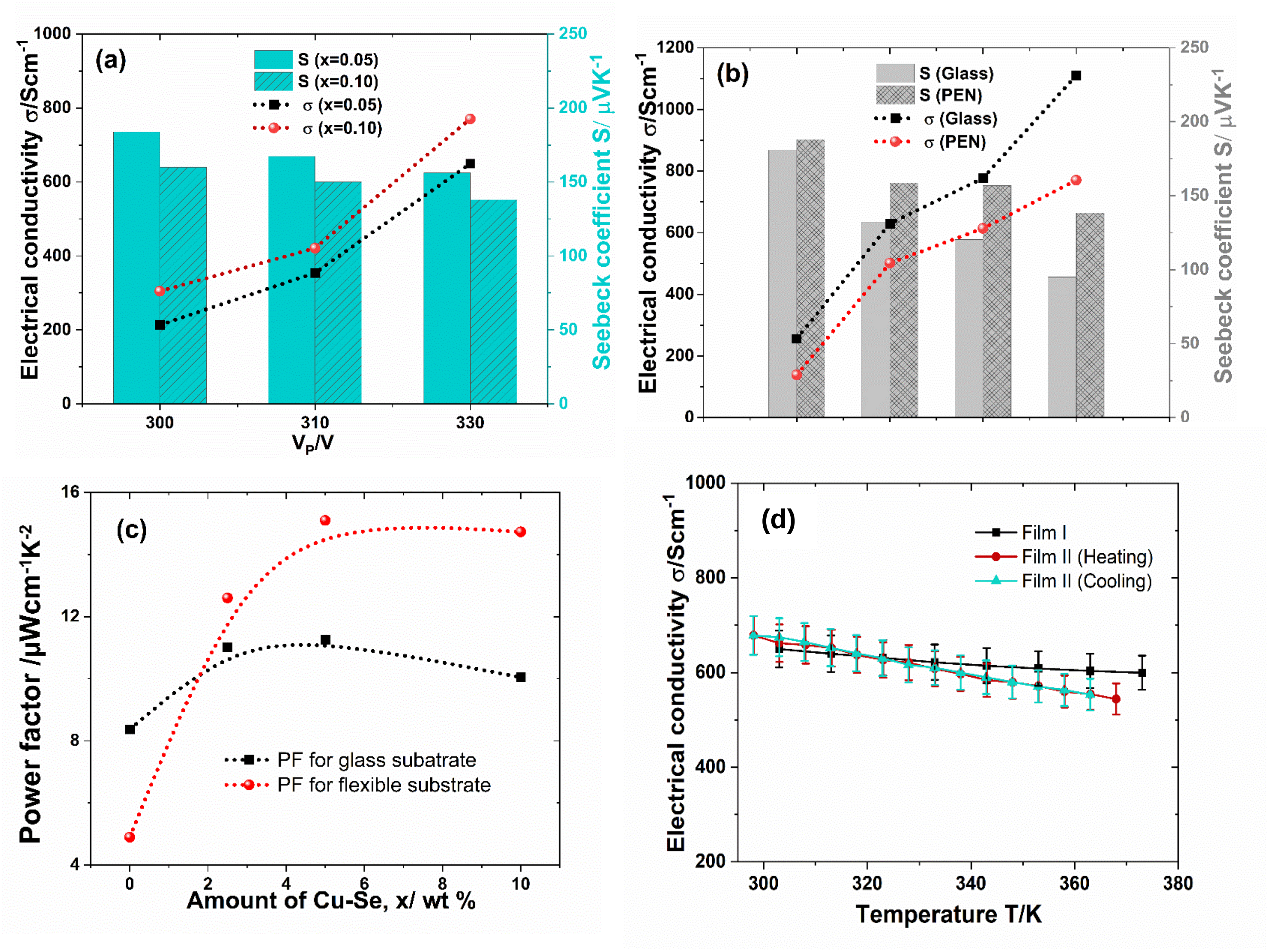

Figure S3: The variation of electrical conductivity σ and Seebeck coefficient S with Vp for the p-BST films for x=0.05 and 0.10 on a flexible substrate (a). The S decreases, and σ increases with increasing Vp. The composition-dependent S, *σ* (b) power factor PF (c) of the p-BST film with x=0.05 on the glass and flexible substrate. The PF on the glass substrate is higher in the pristine film, however, it is higher for the films with x>0 on the flexible substrates. The increase in the electrical conductivity σ of the films is significantly higher on the glass than on the flexible substrate. Most probably, the better compaction of the film due to reaching a higher temperature T> 873 K on the glass during photonic sintering leads to higher mobility and charge carrier concentration p_H increasing σ. The charge carrier concentration p_H is also found to be higher for the printed films on glass for x>0, which might reduce the Seebeck coefficient S. The p_H of the film for x=0.10 on glass is 4.4 $\times 10^{20}$ cm^{-3} with Vp=420 V as compared to 2.6 $\times 10^{20}$ cm^{-3} with Vp=320 V on the flexible substrate.

However, other factors such as mechanical stability and deformation of the substrate during sintering could regulate the transport properties.

A new p-BST TE film with x=0.05 was prepared using a new batch of ink to check the reproducibility and repeatability of its TE performance. The temperature-dependent σ of the films is compared in Figure S3 (d). The results show a similar conductivity value for both films. Furthermore, film II exhibits an insignificant hysteresis in the temperature-dependent σ. The Seebeck coefficient α is also found to be similar, 151 μVK^{-1}.

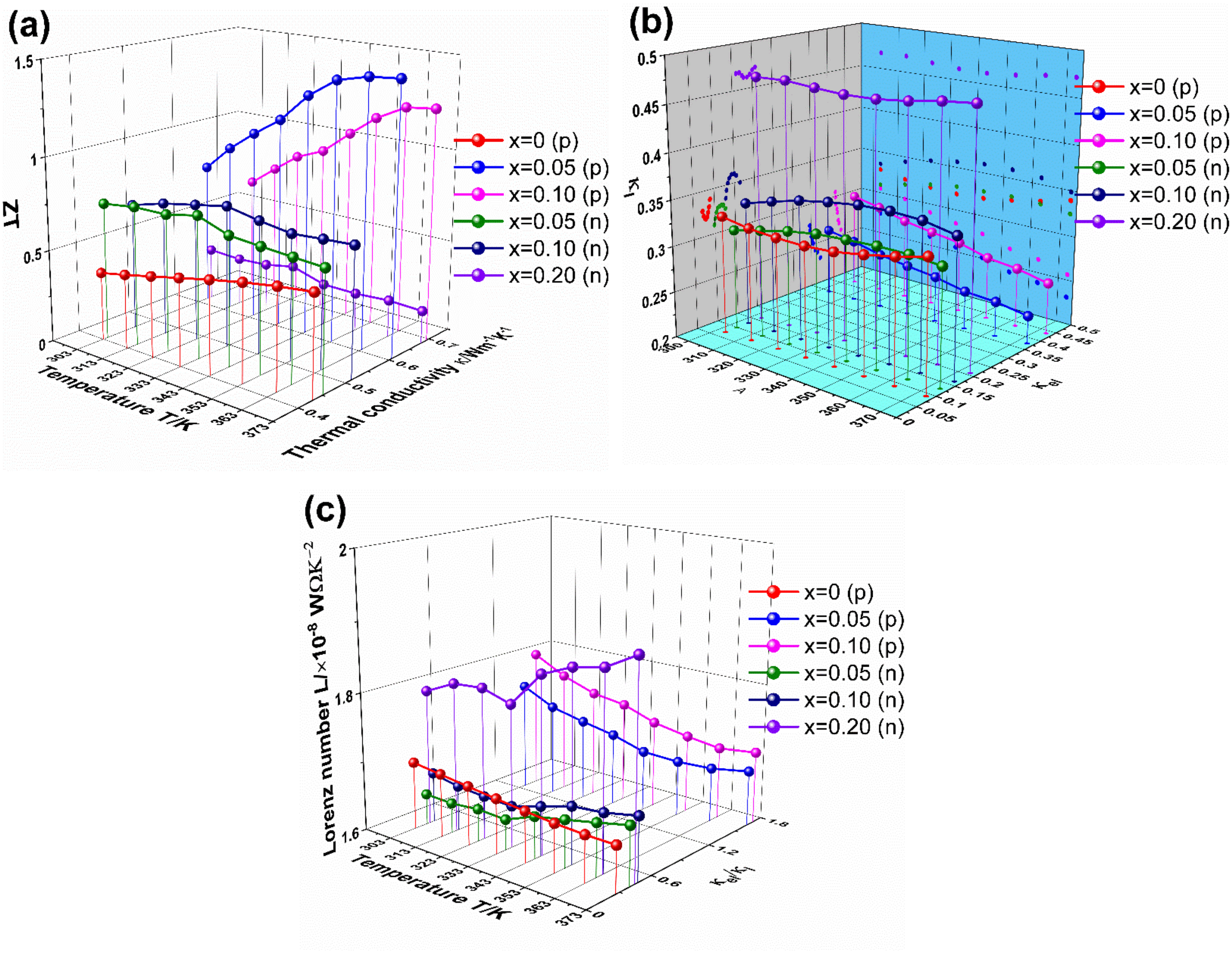

Figure S4: The temperature-dependent thermal conductivity κ and the figure-of-merit ZT of the p-BST and n-BT films (a). The temperature-dependent electronic thermal conductivity κ_{el} to the lattice thermal conductivity κ_l for different x (b). The variation of Lorenz number L and the ratio of the κ_{el} to the κ_l (κ_{el}/κ_l) with temperature for p-BST and n-BT films (c).

The thermal conductivity comprises lattice thermal conductivity (κ_l) and electronic thermal conductivity (κ_{el}). The κ_e is determined using Wiedmann-Franz Law; $\kappa_{el} = \sigma LT$, with L the Lorentz number. The is estimated using the following expressions;[1]

$$L = -\frac{{k_B}^2}{e^2}\frac{(1+\lambda)(3+\lambda)F_\lambda(\eta)F_{2+\lambda}(\eta)-(2+\lambda)^2F_{1+\lambda}(\eta)^2}{(1+\lambda)^2F_\lambda(\eta)^2} \quad (1)$$

where λ is the scattering factor, F the Fermi integral, and η the chemical potential. The η is calculated from the experimental Seebeck coefficient using the expressions;[1]

$$\alpha = -\frac{k_B}{e}\left(\frac{(2+\lambda)F_{1+\lambda}(\eta)}{(1+\lambda)F_\lambda(\eta)} - \eta\right) \quad (2)$$

and $$F_j(\eta) = \int_0^\infty f\varepsilon^j d\varepsilon = \int_0^\infty \frac{\varepsilon^j}{1+e^{(\varepsilon-\eta)}}d\varepsilon \quad (3)$$

where ε is the reduced energy. Assuming λ =0, the L using eqns. (1) – (3). The L is found to be in the range~ 1.6×10^{-8} $W\Omega K^{-2}$ to~ 1.8×10^{-8} $W\Omega K^{-2}$ for all the films indicating degenerate semiconductors (L_{deg} $=1.5\times10^{-8}$ $W\Omega K^{-2}$ to 2.4×10^{-8} $W\Omega K^{-2}$)[2,3]. The lattice thermal conductivity κ_l is calculated via $\kappa_l = \kappa - \kappa_l$. The electronic contribution κ_{el} to κ is found to be only 17 % in the pristine film due to the significant charge scattering by the interfaces and micro-pores. The lattice transport is found to be dominant for all x>0 in n-BT films. The charge carriers govern the thermal transport in the p-BST films for x>0. The ratio κel/κl increases with increasing x and reaches >1 for p-BST films, whereas it does not change significantly for n-BT films. The results indicate that the electronic contribution κel increases significantly due to the reduction of the charge carrier scattering in the p-BST films because of the filling of the micro-pores and soldering of the grains with the high conducting IB. On the other hand, the κ_{el} of the n-BT is not increased substantially with x due to its dissimilar charge carrier polarity to the IB.

The average zT is the parameter of importance for device applications and is given by the equations below;[4]

$$\text{Average figure-of-merit, } \left(zT_{avg}\right) = \frac{1}{\nabla T}\int_{T_c}^{T_h}(zT)dT \quad (4)$$

where T_h, T_c, and $(zT)_{avg}$ are the hot-side temperature, cold-side temperature, and average zT of the films.

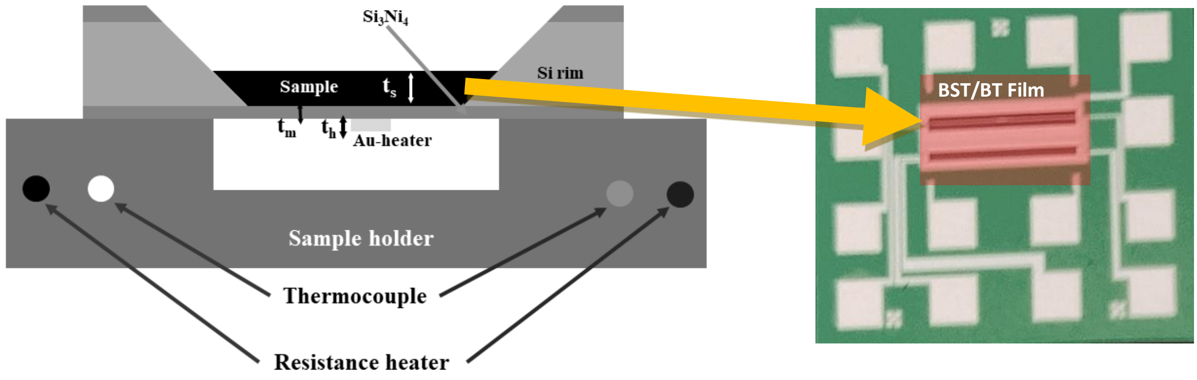

Figure S5. Schematic diagram of the ZT-chips used to measure thermal conductivities of the printed films.

The thermal conductivity of a printed film on a microchip is measured by Linseis TFA (thin film analyzer) system, developed by V. Linseis et. al. [J. Mater. Res., 2016, 31, 20, Phys. Status Solidi A, 2013, 210, 106–118]. The TFA chip is fabricated on a silicon wafer where two thin heaters of 5 µm width are deposited on a free-standing Si3N4 membrane. The membranes is surrounded by an Au rim. The heaters are joined in a 4-wire configuration. A regulated current (I) is applied to the heater; hence a heat flux occurs between the heater and the surrounding silicon. The thermal conductivity is calculated by determining the temperature increase of the heater and applied heating power using a heat flux sensor. The detailed measurement technique and mathematical models are discussed in Ref. J. Mater. Res., 2016, 31, 20.

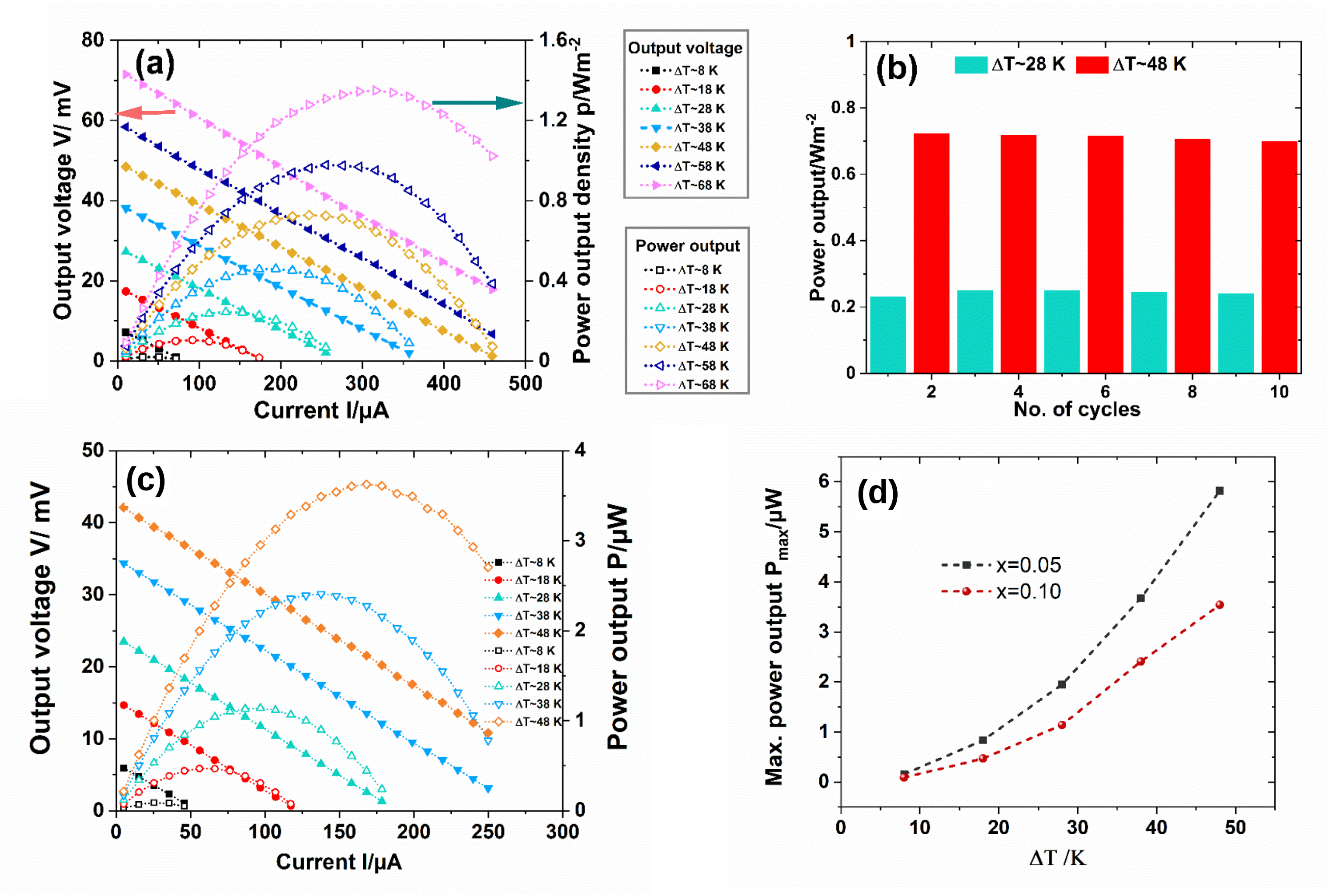

Figure S6: The performance of single-legged f-TEG for x=0.05 (a&b) and 0.10 (c) on PEN substrates. The maximum power output Pmax is higher for x=0.05 than for x=0.10 at all the ΔTs (d).

Reference

1. Xie, H. *et al.* Beneficial contribution of alloy disorder to electron and phonon transport in half-heusler thermoelectric materials. *Adv. Funct. Mater.* **23**, 5123–5130 (2013).
2. Thesberg, M., Kosina, H. & Neophytou, N. On the Lorenz number of multiband materials. *Phys. Rev. B* **95**, (2017).
3. Kim, H. S., Gibbs, Z. M., Tang, Y., Wang, H. & Snyder, G. J. Characterization of Lorenz number with Seebeck coefficient measurement. *APL Mater.* **3**, (2015).
4. Kim, H. S., Liu, W., Chen, G., Chu, C.-W. & Ren, Z. Relationship between thermoelectric figure of merit and energy conversion efficiency. *PNAS* **112**, 8205–10 (2015).
